# Do Working Conditions of Patients in Psychotherapeutic Consultation in the Workplace Differ from Those in Outpatient Care? Results from an Observational Study

**DOI:** 10.3390/ijerph15020227

**Published:** 2018-01-30

**Authors:** Amira Barrech, Reinhold Kilian, Edit Rottler, Lucia Jerg-Bretzke, Michael Hölzer, Monika Annemarie Rieger, Marc Nicolas Jarczok, Harald Gündel, Eva Rothermund

**Affiliations:** 1 Department of Psychosomatic Medicine and Psychotherapy, University Clinic Ulm, 89081 Ulm, Germany; amira.barrech@uni-ulm.de (A.B.); edit.rottler@uniklinik-ulm.de (E.R.); lucia-bretzke@uni-ulm.de (L.J.-B.); marc.jarzcok@uniklinik-ulm.de (M.N.J.); harald.guendel@uniklinik-ulm.de (H.G.); 2 Department of Psychiatry II, University Clinic Ulm, BKH 89312 Guenzburg, Germany; reinhold.kilian@bkh-guenzburg.de; 3 ZfP Suedwuerttemberg, Sonnenbergklinik, 70597 Stuttgart, Germany; hoelzer.michael@sonnenbergklinik.de; 4 Institute for Occupational and Social Medicine and Health Services Research, University Clinic Tuebingen, Competence Centre Health Services Research, Medical Faculty Tuebingen, 72074 Tuebingen, Germany; Monika.Rieger@med.uni-tuebingen.de; 5 Leadership Personality Center Ulm, Ulm University, 89073 Ulm, Germany

**Keywords:** workplace perception, demand-control-support model, depression, health services research, early intervention, help-seeking behavior

## Abstract

In previous studies, it was found that patients treated at a psychosomatic outpatient clinic (PSOC) for common mental disorders showed more severe symptoms than those who used a psychotherapeutic consultation service at the workplace (PSIW). This study examines whether the higher symptom severity of the PSOC patients in comparison to their PSIW counterparts is also related to higher levels of occupational stress as measured by the demand-control-support model (DCS). *N* = 253 participants (PSIW *n* = 100; PSOC *n* = 153) provided self-reported data on demands, decision latitude, social support, and health before consultation. The association between mental health care setting, symptom level and demands, decision latitude, and social support was assessed by means of a path model. Results of the path model indicated that the higher level of depression in PSOC patients was related to higher levels of demands and lower levels of social support. Demands and social support were found to be indirectly associated with treatment setting. No interaction effect between demands, decision latitude, social support, and depression was found. Results of this study reveal that the working conditions influenced the pathway to care process via symptom severity.

## 1. Introduction

Stressful working conditions can have a negative impact on mental wellbeing, and contribute to chronic conditions like mood and anxiety disorders [1,2,3,4,5]. This is indeed estimated to affect 20% of the working population worldwide [6], inciting average costs of around 3.5% of a country’s gross domestic product [7]. Furthermore, affected individuals often experience social damage like the worsening of the social climate among colleagues or with supervisors due to reduced work performance [8], fewer possibilities of participation in the labor market, and an increase of sickness absences [9,10,11,12]. It has been recently shown that 70–80% of persons in need of mental health care in high income countries are not in treatment [13]. It takes an average of six to seven years for a person in need to get adequate treatment [14]. Barriers to early and adequate treatment are diverse, and include personal factors like stigma [15,16,17] as well as contextual factors (e.g., having access to adequate treatment) [18].

Regardless of the beneficial effect that work might have [9,19], unfavorable organizational and societal factors at work are known to determine the interplay between individual mental health and environment [2]. An established theoretical model that explores the influence of the psychosocial work environment on employees’ mental health is the demand-control-support model (DCS) [20,21]. The model posits that working conditions perceived by employees as being characterized by high demands and low decision latitude can negatively affect their mental health [2,22]. The demands of a job encompass aspects like time pressure or workload [23]. Decision latitude describes the extent to which an employee can exert influence over their work (in terms of the possibility of using their skills—skill discretion) and is free to make decisions on work-related tasks such as timing (decision authority) [23]. Moreover, the model postulates that the detrimental effects of high demands and low decision latitude are buffered by social support at work; i.e., they are higher in conditions where employees experience low levels of social support at work. A number of studies have confirmed the detrimental effect of low decision latitude, high demands, and insufficient social support on mental health, as well as the buffering role of social support at work [24,25]. Moreover, social support at work has also been associated directly with mental health [2,22,25]. It has been shown that persistent exposure to high demands and low decision latitude affects mental health permanently, even after the removal of the stressor [22]. However, work and non-work stressors appear to develop differently, and in part independently from each other [4,26].

Psychotherapeutic consultation in the workplace (PSIW) is a new model of care that was implemented in order to reduce the treatment gap and establish close collaboration between regular care and company health promotion [27,28]. Therefore, a standard form of mental health treatment in the German statutory health care system (psychotherapeutic outpatient care in a psychosomatic clinic, PSOC) has been transferred to the workplace setting [27]. Seeking help for mental problems in regular medical care is driven by symptom severity [29,30,31]. However, PSIW allows for an early intervention with less-impaired individuals compared to regular care [30,31].

To date, little is known about the working conditions of those affected by common mental disorders who are seeking help in either a company (PSIW) or clinical setting (PSOC). Considering the fact that individuals taking advantage of the vocational PSIW offer report lower symptom severity [30,31], and based on the propositions of the DCS model [20,21], we hypothesize that these participants also experience lower levels of demands, higher decision latitude, and higher levels of social support at work. We further expect that demands, decision latitude, and social support will be associated with mental health and that social support at work will buffer the negative effect of high demands and low decision latitude on mental health. Hence, the aim of this study was to understand whether help-seeking behavior and working conditions are associated with each other. Therefore, we assessed whether the higher symptom severity of patients treated at the PSOC is also related to unfavorable working conditions in terms of the DCS model.

## 2. Materials and Methods

### 2.1. Study Design and Participants

Data were collected within the study “Psychotherapeutic consultation in the workplace—a new model of care at the interface of company supported mental health care and consultation-liaison psychosomatics”, which is described in detail elsewhere [27], German Clinical Trials Register (DRKS, DRKS00003184). Participants received a short-term psychotherapeutic consultation either in the outpatient care setting (PSOC) or in the workplace setting (PSIW). Treatment was provided by a mental health specialist (psychological or medical psychotherapist) in both settings and was comprised of a diagnostic assessment, first aid, and support in navigating the health care offers or system if needed. The standard treatment form in PSOC and PSIW settings comprises 1–2 sessions.

Participants in the PSOC group were recruited consecutively from two outpatient clinics: University Clinic of Psychosomatic Medicine and Psychotherapy, Ulm, and Sonnenbergklinik, Division of Psychosomatic Medicine of the ZfP, Suedwuerttemberg, Stuttgart (June 2012–January 2013). These patients were mainly referred by general practitioners and some were self-referred.

Participants in the PSIW group were recruited consecutively from November 2011–June 2013 in three companies: an automobile manufacturer, a metal works company, and a security systems company. The employees in two companies were mainly referred to PSIW by the occupational physicians or the social workers. The employees at the third company were mainly self-referred.

**Figure 1:**
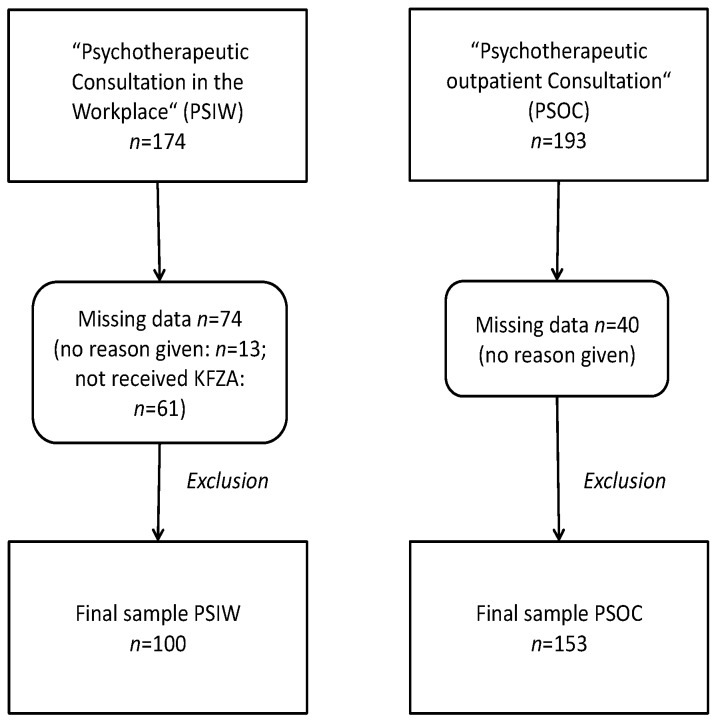
Participant flow chart. KFZA: “Short Questionnaire for Job Analysis”.

To be eligible, participants had to be at least 18 years old and proficient in understanding and writing German. In the PSOC group, individuals without employment were excluded from the data analysis. In one company, there was no consensus among company representatives (management, company health service, work council, and others) about including a measurement regarding working conditions like the Short Questionnaire for Job Analysis (KFZA) [32]. Thus, employees in one company (i.e., 61 participants from PSIW) did not receive the KFZA. We performed list wise deletion for these individuals as shown in Figure 1.

A total of 367 participants provided data (*n*_PSIW_ = 174, *n*_PSOC_ = 193) and defined the source population for the present study. From this population of *n* = 367, participants with missing information on any of the study variables were excluded (*n* = 114), leading to a final sample size of *n* = 253 participants (123 women and 130 men). The final sample consisted of *n*_PSIW_ = 100, *n*_PSOC_ = 153. A drop-out analysis revealed that participants with missing information were significantly older (45 vs. 41 years) and more frequently belonged to the PSOC group (76% vs. 61%). 

The study followed the Declaration of Helsinki and was approved by the Medical Ethics Board of the University Medical Centre of the University of Ulm, 26 September 2011 (Ref No. 224/11). All participants provided written informed consent.

### 2.2. Measurement

All variables were assessed by a self-administered questionnaire.

The DCS model was measured by means of 26 items and 11 scales from the “Short Questionnaire for Job Analysis” (KFZA) [32]. Decision latitude was measured by means of six items (three items for decision authority, e.g., “Can you plan and schedule your work autonomously?”, and three items for skill discretion, e.g., “Can you fully employ your knowledge and skills at work?”) using a five-point Likert scale ranging from 1 (“very little”) to 5 (“a lot”). Demands were measured with six items (e.g., “I am often under time pressure”) using a five-point Likert scale ranging from 1 (“do not agree at all”) to 5 (“agree completely”). Social support at work was measured with four items (e.g., “I can rely on my colleagues if things get difficult at work”) using a five-point Likert scale ranging from 1 (“do not agree at all”) to 5 (“agree completely”). The scales displayed satisfactory reliability (Cronbach’s alpha coefficients for decision latitude = 0.802, demands = 0.748, and social support = 0.776).

Depression was measured using the German version of the patient health questionnaire for depression including nine items (PHQ-9). Interpretation of PHQ-9 scores was based on the diagnostic criteria of the DSM-IV and ICD-10 using the recommended cut-off of 10 or above to distinguish between clinical and nonclinical levels of symptoms [33,34]. The PHQ-9 is a valid (sensitivity 0.88, specificity 0.88) and reliable (internal reliability 0.89, test-retest reliability 0.84) instrument [34].

### 2.3. Statistical Analyses

Descriptive characteristics of the sample were evaluated by using the mean ± standard deviation (SD) or number and proportions, if applicable. Mean differences of continuous variables were assessed by means of *t*-test, mean differences of categorical variables were measured by means of *Χ*^2^-test.

In order to investigate the associations between job demands, social support, decision latitude, depression, and treatment setting, a structural equation model (SEM) without latent variables (path model) was computed containing gender, age, educational status (high vs. low, high educational level was defined as having a university degree) and job status (high vs. low, high job status was defined as having discretionary power) as exogenous variables and job demands, decision latitude, social support, depression, and treatment setting as endogenous variables. Job demands, decision latitude, and social support were centered at their means for the purpose of the interpretation of multiplicative interaction effects. Two-way multiplicative interaction effects between job demands × social support, job demands × decision latitude, social support × decision latitude, and the three-way interaction job demands × decision latitude × social support were tested [35]. For the purpose of mediation analysis, indirect effects were obtained by decomposition of total effects. Maximum likelihood estimators with robust standard errors (MLR) were applied for model estimation [36]. After the estimation of the saturated model including all non-recursive model paths and interaction effects, a restricted model was estimated including only the significant paths to get a parsimonious model. The model fit of the restricted model was tested by means of the Akaike information criterion (AIC) and the Bayes information criterion (BIC). Both criteria allow the comparison of nested structural equation model (SEM), with lower values indicating a better model fit [36].

Path coefficients b, their standard error (S.E.) and the respective *p*-value are reported. *R*^2^ for the continuous and pseudo *R^2^* [37] for the categorical endogenous variables are reported.

The level of significance was set to *p* ≤ 0.05. Path analyses were conducted with MPLUS8 (Version 8 [36], Muthen & Muthen, Los Angeles, CA, USA), and all other statistical analyses were performed using SPSS version 23 for Mac (IBM Corp., Chicago, IL, USA).

## 3. Results

A total of 367 participants provided data (*n*_PSIW_ = 174, *n*_PSOC_ = 193) and defined the source population for the present study. From this population of *n* = 367, participants with missing information on any of the study variables were excluded (*n* = 114), leading to a final sample size of *n* = 253 participants. Of these, 48.6% were women (*n* = 123) and 51.4% were men (*n* = 130). The final sample consisted of *n*_PSIW_ = 100, *n*_PSOC_ = 153. A drop-out analysis revealed that participants with missing information were significantly older (45 vs. 41 years), significantly more often without a university degree (83% vs. 73%), and more frequently belonged to the PSOC group (76% vs. 61%).

### 3.1. Descriptive Results

Two hundred and fifty-three participants were included in the analyses (51.4% male). Of the total sample, 39.5% (*n* = 100) received therapeutic treatment via a consultation at the workplace (PSIW) and 60.5% (*n* = 153) via outpatient care (PSOC). Table 1 provides an overview of the descriptive characteristics of the sample. Mean age of the sample was 41.21 ± 11.47 years (range: 18 to 63 years), the mean level of depression was 13.18 ± 6.48 (range: 0–27), the average level for decision latitude was 6.65 ± 1.74 (range: 2–10), it was 2.95 ± 0.93 (range: 1–5) for demands, and the average level of social support at work was 3.25 ± 0.97 (range: 1–5). Participants in the PSIW group were significantly older (45 vs. 38 years) and more often male (75% vs. 36%).

### 3.2. Results of the Structural Equation Model

**Figure 2:**
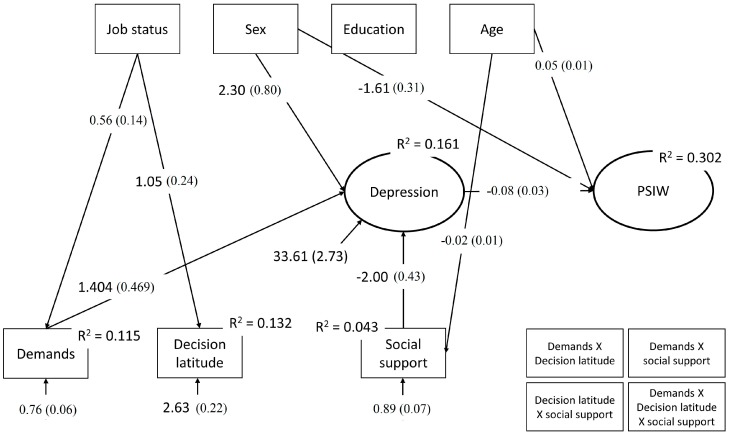
Significant non-standardized path coefficients of the saturated structural equation model (SEM).

Figure 2 shows the significant non-standardized path coefficients of the saturated SEM.

None of the tested interactions (social support × demands: *b* = −0.16, *se* = 0.48, *p* = 0.738; demands × decision latitude: *b* = 0.11, *se* = 0.23, *p* = 0.620; decision latitude × social support: *b* = 0.16, *se* = 0.20, *p* = 0.435; demands × decision latitude × social support: *b* = 0.31, *se* = 0.21, *p* = 0.153) were significant.

The path coefficients to the treatment setting indicate that job demands (*b* = −0.32; *se* = 0.18; *p* = 0.083), decision latitude (*b* = 0.04; *se* = 0.09; *p* = 0.679), and social support (*b* = 0.22; *se* = 0.18; *p* = 0.225) were not significantly directly associated with treatment setting. However, decomposition of total effects indicated that job demands were indirectly negatively associated (*b* = −0.11; *se* = 0.05; *p* = 0.038) and social support was indirectly positively associated (*b* = 0.151; *se* = 0.06; *p* = 0.010) with treatment setting via depression, while decision latitude was not (*b* = 0.02; *se* = 0.02; *p* = 0.318). Moreover, significant direct associations with depression, age, and gender were found. Participants who were treated at PSIW were less depressed than those treated at PSOC (*b* = −0.08; *se* = 0.03; *p* = 0.003; OR = 0.927), and with increasing age participants had a greater probability of being treated by PSIW (*b* = 0.05; *se* = 0.01; *p* = 0.002; OR = 1.046). Female participants were less likely to be treated in PSIW than their male counterparts (*b* = −1.61; *se* = 0.31; *p* = 0.000; OR = 0.200). No significant direct effects were found for educational level (*b* = −0.06; *se* = 0.39; *p* = 0.882) or job status (*b* = 0.18; *se* = 0.38; *p* = 0.642).

Path coefficients for the endogenous variables further indicate significant direct effects for job demands and social support to depression. While depression increased with increasing job demands (*b* = 1.40; *se* = 0.47; *p* = 0.003), it decreased with increasing social support (*b* = −2.00; *se* = 0.43; *p* = 0.000). Decision latitude had no significant effect on depression (*b* = −0.27; *se* = 0.25; *p* = 0.278). From the endogenous variables, female participants were more depressed than their male counterparts (*b* = 2.30; *se* = 0.80; *p* = 0.004), while age (*b* = −0.01; *se* = 0.04; *p* = 0.870), educational level (*b* = 0.15; *se* = 0.90; *p* = 0.426), and job status (*b* = −0.70; *se* = 0.87; *p* = 0.426) were not.

Path coefficients for the exogenous variables indicate that with increasing age participants reported less social support (*b* = −0.02; *se* = 0.01; *p* = 0.007), but social support was not related to sex (*b* = 0.15; *se* = 0.12; *p* = 0.214), educational level (*b* = 0.20; *se* = 0.14; *p* = 0.149), or job status (*b* = 0.02; *se* = 0.14; *p* = 0.914). Participants in management positions perceived more job demands (*b* = 1.05; *se* = 0.24; *p* = 0.000) and more decision latitude (*b* = 0.56; *se* = 0.14; *p* = 0.000). Age was neither related to demands (*b* = 0.00; *se* = 0.01; *p* = 0.933) nor to decision latitude (*b* = 0.00; *se* = 0.01; *p* = 0.769). Demands were also not associated with sex (*b* = −0.08; *se* = 0.12; *p* = 0.493) or educational level (*b* = 0.12; *se* = 0.15; *p* = 0.429). Decision latitude was also not associated with sex (*b* = 0.41; *se* = 0.21; *p* = 0.051) or educational level (*b* = 0.34; *se* = 0.24; *p* = 0.19).

As indicated by the *R*^2^ for the endogenous variables, the model explained 11.5% of the variance in job demands, 4.3% of the variance in social support, 13.2% of the variance in decision latitude, 16% of the variance in depression, and 30% of the variance of the logits of being treated at PSIW.

The model fit test for the restricted in comparison to the saturated model revealed that the model including only the significant model paths had a lower AIC (4238.807 vs. 4263.813), a lower BIC (4302.408 vs. 4405.148), and a lower adjusted BIC (4245.345 vs. 4278.341), indicating an improved model fit for the restricted model.

## 4. Discussion

This is the first study to assess the role of working conditions in the access to treatment for common mental disorders via consultation in the workplace (PSIW) versus access to treatment in an outpatient care facility (PSOC). Experiencing high demands was indirectly associated with a lower likelihood of accessing mental health care treatment via PSIW, while reporting high social support was associated with a higher likelihood hereof. This effect was mediated by depression. Accessing mental health care via PSIW was directly associated with male sex, older age, and lower levels of depression.

These findings partly confirm our hypotheses. The mediating effect of depression suggests that participants accessing therapeutic help via the PSOC group might have previously experienced higher levels of demands and subsequently were at a higher risk of developing depressive symptoms, which in turn may have led to help-seeking in a clinical setting. Though the cross-sectional nature of the present study does not allow for conclusions as to causality, these results are generally in line with previous findings from prospective studies which have established an association between occupational stress and the subsequent onset of depressive symptoms [2,24].

Contrary to the assumption of the DCS model and in line with previous findings [38,39], decision latitude, the interaction between demands and decision latitude, and the interaction between demands, decision latitude, and social support were not associated with either depression or pathway to treatment. There is debate as to whether the components of the DCS model exert an additive (main effects for the three dimensions) or multiplicative (i.e., interactive) effect [40]. Both were tested in the present sample, but only main effects of demands and social support on depression were found. A possible explanation for this may lie in the match regarding the assessment of the demands dimension with that of the decision latitude dimension in the model: if the assessment of decision latitude (e.g., autonomy with regard to timing of tasks) does not measure the same domain of demands (e.g., emotional demands), there may not be a buffering effect of decision latitude on the association between job demands and mental health [38]. This may also explain the non-significant moderating role of social support on the association between demands, decision latitude, and depression. A further explanation for the non-significant finding could lie in the measures used to assess decision latitude and demands: it is imaginable that the questions may not be relevant in all types of work in the same manner (e.g., industrial setting vs. service sector) and therefore do not capture the constructs adequately [41]. Moreover, in their recent meta-analysis on moderating effects on the relationship between demands, decision latitude, and social support, Fila et al. found that perceptions of the relationships between the three components of the model differ by occupation group [42]. It is therefore also imaginable that a homogeneity in terms of occupations in the present sample may have influenced findings. However, this is not verifiable in the present sample, as information on occupation type is not available.

In line with previous findings, social support was found to be directly associated with lower levels of depression and indirectly associated with a higher likelihood of accessing mental health care via PSIW. It is well established in the literature that there is a relationship between social support at work and mental and physical health outcomes, including both self-reported and objective health measures [25,43].

Moreover, previous findings for the sample revealed that participants in the PSIW group were less impaired with regard to work ability, quality of life, and mental health [30,31]. Our results are in line with data from a longitudinal study of workers in Sweden. The authors revealed that work characteristics such as poor job satisfaction are associated with later care-seeking [44]. Our findings support the usefulness of offering PSIW in order to address mental health issues at an early stage, prior to chronification.

Some limitations need to be considered. First, the cross-sectional character of the study does not allow any causal interpretation. Second, causal interpretation of the associations is impossible because the access to PSIW was limited to employees of the participating firms. Moreover, there was no possibility of assessing whether participants in the PSOC sample had the opportunity to attend a psychotherapeutic consultation in their workplace. Therefore, the presented results provide only an explorative background for the generation of hypotheses on the role of demands, decision latitude, social support, and depression in the pathway to psychiatric care. Third, the fact that 61 participants from PSIW did not receive the KFZA due to company policies might have introduced bias. Fourth, a drop-out analysis revealed that excluded participants were older, had a lower educational level, and were more often from the PSOC group, which possibly led to an underestimation of effects (e.g., treatment via PSIW was associated with older age, and less social support was associated with older age). However, controlling for these variables in the structural equation model may have partly compensated for the differences. Fifth, the sampling procedure (i.e., only individuals seeking mental health care) and the voluntary nature of study participation may limit the generalizability and representativeness of the sample. By controlling for age, sex, education, and job status in all inferential analyses, the effect of selection bias issues was reduced to a certain extent. Nevertheless, further studies with random sampling are needed to further generalize the present findings.

## 5. Conclusions

In conclusion, the present study suggests that patients accessing psychotherapeutic treatment via PSIW may not yet be exposed to high levels of demands, compared to those being treated via PSOC. The higher level of depressive symptoms in patients treated by the psychosomatic outpatient clinic in comparison to those using psychotherapeutic counselling in the workplace is related to higher levels of demands and lower levels of social support. Our findings support the idea that the workplace can be a pivotal social context to address mental health problems early and therefore stop social damage that is the manifestation of a downwards spiral of problems due to reduced work performance and chronic conditions. These results underline the important role of the workplace environment in mental health promotion. Further studies are needed to investigate the effectiveness of providing psychotherapeutic consultation in the workplace in order to reduce occupational stress and improve social support in order to prevent mental disorders.

## Figures and Tables

**Table 1:** Descriptive characteristics of the sample (*n* = 253).

Characteristic	Total Sample % (*n*)/Mean ± SD	PSOC (*n* = 153) % (*n*)/Mean ± SD	PSIW (*n* = 100) % (*n*)/Mean ± SD
Age	41.21 ± 11.47	38.94 ± 11.65	44.66 ± 10.32
Male sex	51.38 (130)	35.95 (55)	75.00 (75)
Depression (PHQ-9)	13.18 ± 6.48	14.50 ± 6.47	11.17 ± 5.98
Decision latitude	6.65 ± 1.74	6.63 ± 1.74	6.70 ± 1.75
Demands	2.95 ± 0.93	3.00 ± 0.96	2.87 ± 0.88
Social support	3.25 ± 0.97	3.22 ± 1.04	3.29 ± 0.85

SD: standard deviation. PSOC: psychosomatic outpatient clinic. PSIW: psychotherapeutic consultation service at the workplace. PHQ-9: patient health questionnaire for depression including nine items.
